# Transcription factor co-localization patterns affect human cell type-specific gene expression

**DOI:** 10.1186/1471-2164-13-263

**Published:** 2012-06-21

**Authors:** Dennis Wang, Augusto Rendon, Willem Ouwehand, Lorenz Wernisch

**Affiliations:** 1 MRC Biostatistics Unit, Institute of Public Health, Robinson Way, Cambridge, UK; 2 Department of Haematology, University of Cambridge, Long Road, Cambridge, UK

**Keywords:** Transcriptional regulation, Gene expression, ChIP-Seq, Regression modeling

## Abstract

**Background:**

Cellular development requires the precise control of gene expression states. Transcription factors are involved in this regulatory process through their combinatorial binding with DNA. Information about transcription factor binding sites can help determine which combinations of factors work together to regulate a gene, but it is unclear how far the binding data from one cell type can inform about regulation in other cell types.

**Results:**

By integrating data on co-localized transcription factor binding sites in the K562 cell line with expression data across 38 distinct hematopoietic cell types, we developed regression models to describe the relationship between the expression of target genes and the transcription factors that co-localize nearby. With K562 binding sites identifying the predictors, the proportion of expression explained by the models is statistically significant only for monocytic cells (p-value< 0.001), which are closely related to K562. That is, cell type specific binding patterns are crucial for choosing the correct transcription factors for the model. Comparison of predictors obtained from binding sites in the GM12878 cell line with those from K562 shows that the amount of difference between binding patterns is directly related to the quality of the prediction. By identifying individual genes whose expression is predicted accurately by the binding sites, we are able to link transcription factors *FOS*, *TAF1* and *YY1* to a sparsely studied gene *LRIG2*. We also find that the activity of a transcription factor may be different depending on the cell type and the identity of other co-localized factors.

**Conclusion:**

Our approach shows that gene expression can be explained by a modest number of co-localized transcription factors, however, information on cell-type specific binding is crucial for understanding combinatorial gene regulation.

## Background

Cellular development requires the precise control of gene expression states. Transcription factors (TFs) are involved in this regulatory process through their combinatorial binding to DNA. Although one factor may be identified as the global regulator of a cell differentiation process, it often works together with other TFs in complexes to achieve precise control of expression at different loci [1]. By coupling high-throughput sequencing technologies with chromatin immunoprecipitation (ChIP-Seq), recent studies have identified thousands of regions along the human genome where TFs co-localize to interact and form gene regulatory complexes [2-4]. However, such binding information for any given TF is only available for a limited number of cell types. It is therefore of great theoretical as well as practical importance to ask how far differences in binding patterns between cell types limit our ability to use TF binding patterns from one cell type to explain gene expression in another. Can we infer a universal regulatory network from TF binding experiments in a small range of cell types, so that by using just TF expression data we can predict gene expression in other cell types? If this turns out to be difficult, the inference of a universal gene regulatory network for a broad range of cell types from just, say, expression data without detailed knowledge of actual TF binding in each cell type might be impossible.

A variety of computational and experimental approaches can be used to measure the effect of co-localized TFs on a single target gene [5]. One way is by exploring the transcriptome over many different conditions, in order to relate gene expression changes to varying levels of TF expression [6]. To assess this relationship between TFs and genes, we can measure how well the TF expression levels predict the expression levels of genes whose promoters they bind. Quantitative models have been useful for predicting the level of gene expression given a limited set of predictors [7]. By assuming that TF binding sites are conserved in the genome of an organism, these models described gene expression as a function of TF binding affinity, which they estimated using TF expression and TF binding motifs [8-10]. However, by using predicted TF binding sites rather than observed sites, these models achieved limited accuracy at predicting human cell type-specific expression changes. Some more recent models have used binding sites of multiple TFs observed through ChIP-Seq [11-13], but they estimate TF binding affinity only for the cell type used in ChIP-Seq, and therefore, cannot predict gene expression in other cell types. Furthermore, no existing model incorporates information about the co-localization of TF binding sites, which may help reduce the number of non-relevant TF predictors in the model.

In this study, we used a reduced set of co-localized TFs close to a target gene to select TFs as predictors in a nonlinear regression model for predicting the expression of the target gene from TF expression levels. Regression coefficients for the nonlinear regression models were then inferred from gene expression data across different cell types and conditions. Expression prediction models were tested for the amount of expression prediction accuracy that can be achieved across 38 distinct cell types and 32 major tissues, given that TF binding information for the selection of predictors came from only the K562 cell type.

As potential predictors we used TFs known to regulate a wide range of cellular processes (Additional file 1: Table S1). For example, *GATA2* and *NFE2* are known to be involved in the control of hematopoiesis [14,15], while two widely studied oncogenes, *MYC* and *FOS*, are more ubiquitous TFs [16]. For each gene, a nonlinear regression model describing the gene’s expression level is constructed using only TFs that have binding sites co-localized within the gene’s promoter region. In accordance with common terminology, we define these regions of binding site co-localization as cis-regulatory modules (CRMs). The regression models account for both linear and non-linear effects as well as interactions between TFs. In the case of *YY1*, the TF is known to co-regulate genes, but may itself be regulated by other TFs [17,18]. The models we show describe combinatorial functions for each set of co-localized TFs, which allowed us to determine whether a TF, such as *YY1*, enhances, represses, or has no effect in the presence of other TFs. We identified many interacting pairs of TFs not known to co-operatively work together, presumably because they were previously examined in a limited number of cell types or using only linear regression methods.

We took the prediction accuracy of a regression based on TFs in a particular CRM for a target gene as an indicator of how much influence this CRM exerts over the target. This allowed us to analyse the relationship between the features of CRMs, such as their TF composition, and their effect on target genes. We compared CRMs from ChiP-Seq data on the GM12878 cell line with equivalent CRMs from the K562 cell line for differences in binding TFs, and the effect of these differences on prediction accuracy. Our analysis shows how far the extensive collection of gene expression and genome-wide TF binding data across multiple human cell types can be exploited to provide valuable insight about regulators of cell differentiation. It also shows the limitations if binding data are available only for a restricted set of cell types and conditions.

## Methods

### ChIP-Seq data

We obtained ChIP-Seq data of 14 TFs in the K562 cell line and 11 TFs in the GM12878 cell line from the ENCODE consortium [3]. Each data set contains a signal map of ChIP DNA fragments, where the signal height is the number of overlapping fragments at each nucleotide position in the genome (NCBI build 36). Enrichment of genomic regions for protein binding was tested against a set of input DNA control (p-value ≤0.01). Peaks indicating regions with sufficient signal above peak height threshold (false discovery rate < 0.05) were identified using the PeakSeq algorithm [19]. After conducting a genome-wide scan for peaks, we examined tracks of putative binding sites for each of the 14 TFs (Additional file 1: Table S1). The position of each binding site was defined by the center of each ChIP-Seq peak. Adjacent binding sites that are within 500bp of each other are grouped. Groups with peaks from two or more different TFs are defined as CRMs. We mapped a CRM to a gene if the centre of the CRM is within 1kb of the gene’s transcription start site (TSS). All distances reported are from the centre of the CRM to the TSS. Non-specific binding of TFs may cause ChIP-Seq to detect randomly distributed binding signals. To reduce false positives we check that a particular combination of TF binding sites occurs more often than in randomly generated CRMs. Random CRMs are generated by resampling (1000 times without replacement) random TFs to replace the original TFs at binding sites in each CRM. The positions of gene TSS are the same as those used in the mapping microarray probe sets to genes. Differences in TF binding between CRMs was measured by the Hamming distance, which is the number of TFs that appear in one CRM but not the other. The Jaccard distance, which is a ratio between the Hamming distance and the number of TFs binding in either CRM, is also the proportion of TFs that are different between two CRMs.

### Gene expression data

Cell type specific patterns of mRNA expression were extracted from the Genome Novartis Foundation SymAtlas data set [20], which measured 79 human tissue samples and cell lines (2 replicates each) using Affymetric GeneChip HG-133U arrays [21]. The gene expression profiles of 38 distinct populations of human hematopoietic cells were from the Broad Institute DMap Project [22]. K562 and GM12878 gene expression data were generated by Ernst *et al.* (2011) [23]. Quantile normalization was applied across expression arrays and the log expression intensities for each gene was mean centered. Probe sets were mapped to a gene’s TSS via transcript identifiers and probe set annotations provided by the Ensembl database (release 54). For cases where there are more than one probe set mapping to a gene’s TSS, the probe set with the most variable expression profile was accepted. In total, 13916 genes were profiled in the data sets, but RNA genes and other non-protein coding genes were not included in our analysis. For more information on the expression profiles of the co-localized TFs, see Additional file 1.

### Expression prediction model

We employed a simple and flexible modeling framework to describe the relationship between co-localized TFs and target genes [24]. The generalized additive model (GAM) is interpretable, because each predictor term is simply the expression of a single TF which occupies a CRM. The GAM implementation in the R package ”mgcv” provides the option of smoothing spline functions for each predictor term, which gives us the flexibility of incorporating non-linear relationships between TFs and genes [25]. For each gene-CRM pair, we considered a model with one or more additive functions:

(1) $$E(y_{i})=\beta_{0}+\overset{n}{\underset{j=1}{\sum }}s_{j}(x_{\mathrm{ij}})+\underset{1\leq j<k\leq n}{\sum }s_{\mathrm{jk}}(x_{\mathrm{ij}},x_{\mathrm{ik}})$$

where *E*(*y*_*i*_) is the expected log expression of the target gene in cell type *iβ*_0_ is the mean expression set to zero, *x*_*ij*_is the log expression of TF *j* in cell type *in* is the number of TFs in the CRM, and *s*_*j*_ is a spline function, where the degree of smoothing is chosen by cross validation in the mgcv package. As opposed to using linear predictors, the estimated non-parametric function can reveal non-linearities in the effect of TF on target gene. In this model we also allow for second-order interactions where *s*_*jk*_(*x*_*ij*_*x*_*ik*_) is now a set of unknown partial bidimensional functions. This accounts for possible interactions between TFs in a CRM, whereby a TF’s effect on gene expression varies according to the effect of another TF in the CRM. To ensure that each model does not simply describe the mean expression level of a target gene, we mean centered all the expression profiles.

### Training and testing

Every gene with a CRM located within its promoter region (1kb around the TSS) was tested for expression prediction. For each CRM-gene pair, we inferred the parameters *β*_0_, *s*_*j*_ and *s*_*jk*_ for the regression equation above using the expression profiles *x*_*ij*_ of the co-localized TFs *j* and *y*_*i*_ of the gene across samples *i*∈*S*_*T*_ from the training set *S*_*T*_. We then predicted gene expression across the samples *i*∈*S*_*P*_in the test set *S*_*P*_ using the TF expression *x*_*ij*_in those samples as predictors. The prediction step gave us a predicted gene expression value ${ŷ}_{i}$ for each target gene in a sample *i*∈*S*_*P*_. The prediction accuracy was then measured by calculating the square of the Pearson correlation coefficient (denoted by *R*^2^) between the predicted expression ${ŷ}_{i}$ and the observed expression *y*_*i*_for all samples in *S*_*P*_. 5-fold cross-validation was performed to assess how well predictions for each gene would generalize to new sample data sets. The *R*^2^ statistics reported were averaged over the cross-validations. When predicting expression variation across a single cell types, leave-one-out cross-validation was performed. The *R*^2^statistic in this case is the square correlation coefficient between the predicted ${ŷ}_{g}$ and observed *y*_*g*_ for a single cell type in *S*_*P*_across genes *g*∈*G*, where *G* is the set of all genes with CRMs in their promoters. This involved training the models on expression profiles from all cell types except for one and then predicting gene expression for the left-out cell type. Since each cell or tissue type has two biological replicates, we used the average *R*^2^between replicates. We used the Wilcoxon rank sum test to compare the *R*^2^ and the squared prediction error statistics between models generated by different CRMs.

### Assessing expression prediction significance

The *R*^2^statistic for prediction by any given model may be biased by the correlation between expression profiles in the training set and test set. Therefore, we tested to see if the observed co-localized TFs predict target gene expression better than any other set of TFs. For each gene expression model, we produce two types of null expression models with the same number of predictors, but we resampled the TFs. In one type of null model, instead of using the TFs observed to be in the CRM, we randomly resample from the 14 different TFs found in K562. For the second type of null model, we randomly resample TFs from a larger set of 41 different TFs identified as important for regulators of hematopoiesis [22]. A TF is resampled only if it is expressed in K562 cells, and if its binding sequence motif is enriched (p-value <10^−7^) in the target gene (within 1kb of the transcription start site). Further details about the six different motif-finding methods and a motif-clustering pipeline used by Novershtern *et al.* can be found from the Broad DMap Project (http://www.broadinstitute.org/dmap). Finally, we use the 1000 replicates of null models to bootstrap each *R*^2^ statistic generated by our expression models. This gives us a p-value for a how well the observed co-localized TFs in a CRM predicts target gene expression compared to other possible sets of TFs.

## Results

### High number of TF co-localization hot-spots along the genome

**Figure 1:**
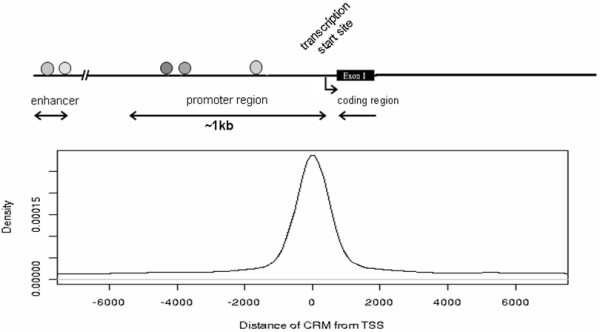
**TF binding distribution.** Adjacent TFs are grouped into CRMs if they lie within 500bp of one another. A high proportion of CRMs (19%) are concentrated within 1kb of a gene TSS.

In order to gain insight into the distribution of TF co-localization across the human genome, we first analyzed the ChIP-Seq data of 14 TFs from the K562 cell line [3]. Peaks for DNA fragment enrichment in each data set are signals along the genome for where a specific TF binds. By comparing all TF binding sites in a cell type, we identified 37529 regions where two or more TFs co-localize within 500bp of each other. As shown in Figure 1, a high concentration of co-localizations lie within 1kb of the transcription start site (TSS). We defined regions of TF co-localization as CRMs, because they are in the vicinity of gene promoters and may have regulatory potential. Each CRM contains different combinations of co-localized TFs. In the K562 cell, we identified 9665 CRMs containing two or more bound TFs, and with 1051 different binding combinations (Additional file 2: Data set). We noticed that 355 unique combinations of co-localized TFs have a probability of less than 1×10^−4^ of occurring as many times by chance. All known pairs of interacting TFs (Additional file 1: Figure S2) appear in significant combinations. Only such frequently occurring combinations of TFs in CRMs were analyzed further to determine if they have potential to influence target gene expression.

### Co-localized TFs predict target gene expression

We examined 6582 genes in the K562 cell type with CRMs positioned within their promoter region (± 1kb from the TSS), and generated expression prediction models for every CRM-gene pair. A CRM is mapped to a gene if at least one of their TF binding sites is located within the promoter. The co-localized TFs in each CRM are the explanatory variables used in each model. We assessed whether a CRM is an active gene regulatory region by how well its TFs can predict the expression of target genes.

#### High accuracy of expression prediction for single samples

**Figure 2:**
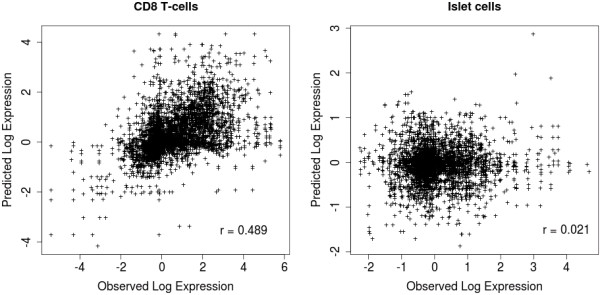
**Predicted vs observed expression.** Predicted versus observed fold changes in gene expression for peripheral blood T-cells and pancreatic islet cells. We predicted expression for all genes with co-localized TFs at their promoters (within 1kb of TSS).

We analyzed the K562 ChIP-seq data to identify the co-localized TF binding sites, and assessed the presence of active gene regulatory regions across 79 different tissue and cell samples from the Novartis SymAtlas data set. The expression predictions, as shown in Figure 2, correlated well with the observed gene expression in CD8 T-cells, but less well for pancreatic islet cells. The models predict gene expression more accurately for samples with a single cell type compared to samples composed of many different cell types (Additional file 1: Table S2). Given that TF expression levels and TF-TF interactions are cell type-specific [21,26], the expression prediction models are expected to be less accurate for heterogenous samples. The prediction accuracy for our models also differs depending on the cell type in each sample. Compared to other cell types, we observed a significantly higher expression prediction accuracy (p-value =1.9×10^−10^) for samples containing blood cells or certain neuronal cells. Since the prediction accuracy measure is an indicator of how likely the CRM is an active gene regulatory region, the results suggest that K562 CRMs may only be active in cell types of a common lineage or with similar phenotypic characteristics.

#### CRMs explain cell type-specific expression

**Figure 3:**
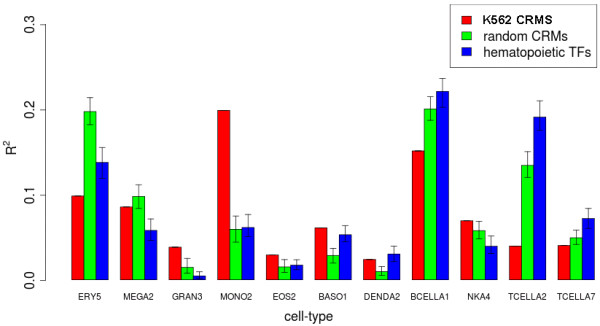
**Expression prediction accuracy across terminally differentiated hematopoietic cells.** Comparison of *R*^2^between observed and predicted expression in hematopoietic cell populations; erythroid cells (ERY5), megakaryocytes (MEGA2), neutrophils (GRAN3), monocytes (MONO2), eosinophils (EOS2), basophils (BASO1), myeloid dendritic cells (DENDA2), naive B cells (BCELLA1), mature NK cells (NK4), Naive CD8+ T cells (TCELLA2), and CD4+ effector memory T cells (TCELLA7). *R*^2^from expression models generated from K562 CRMs (red) are compared to models with TFs randomly selected from the 14 in K562 (green), and models generated from binding motifs of 41 TFs important to hematopoiesis (blue). Bars show 95% confidence intervals for the null models.

Since the prediction accuracy for gene expression models is high for blood cell samples in the Novartis SymAtlas, we further assessed differences in prediction accuracy when the gene expression models were applied to distinct hematopoietic cell types (DMap data set) [22]. Co-localized TFs in K562 CRMs were used once again to define the explanatory variables in each gene expression model. We analyzed the predictions made for gene expression in each terminally differentiated hematopoietic cell type (Figure 3). The models show that K562 CRMs explain a higher proportion of expression for B-cells and monocytes compared to other cell types. The high proportion of explained variation may be due to by chance correlation in expression between genes and TFs, or due to low variation in expression for many of the genes. Hence, we tested whether a specific set of TF expression profiles predicts a specific target gene’s expression better than a random set of TFs by comparing the *R*^2^ from our gene expression models to the *R*^2^from null models. One type of null model was generated by replacing each TF occupying a CRM by a randomly selected one from the list of 14 TFs found in K562. The other null model was generated from TFs that were identified as important for hematopoiesis and which have binding motifs enriched in the target gene [22]. The null models based on binding motifs show that there are other TFs not profiled in K562 which may be important regulators in other cells, like HSC and T-cells. Only for monocytic cells did the expression prediction models, generated from K562 CRMs, explain significantly more variation (p-value <0.001) than the two null models. It has been reported that K562 cells have characteristics similar to early-stage monocytes [27], and their gene-TF relationships may too be similar.

### Dissimilar CRMs have different regulatory activities

**Figure 4:**
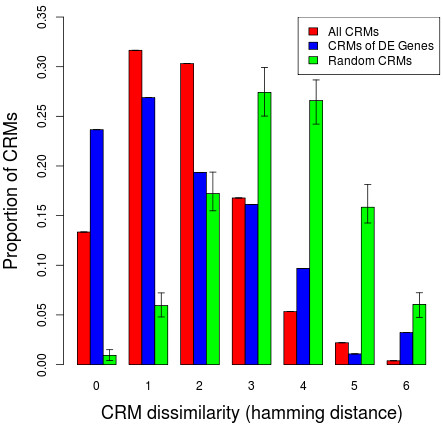
**Distribution of dissimilar CRMs at differentially expressed genes.** The dissimilarity between CRMs at the promoter regions of all genes (red bars) and at the promoter regions of differentially expressed genes (blue bars) are compared to the dissimilarity between randomly generated CRMs (green bars with 95% confidence intervals). Dissimilarity between an overlapping pair of CRMs is described by the number of different TFs (Hamming distance) bound to a K562 CRM compared to a GM12878 CRM.

We assessed how robust the TF co-localization patterns are across the different cell types. From the ChIP-Seq data for the K562 cell line and the GM12878 cell line, we compared the binding profiles of 11 TFs assayed in both cell lines. Of the 12763 CRMs detected along the K562 genome and 12824 CRMs in GM12878, 3450 of the regions in both cell types overlap. Two CRMs are considered to overlap if the intervals of the CRMs both overlap by more than 50%. Within these overlapping CRM regions, we examined the dissimilarity of TF binding site profiles between the cell types. The dissimilarity between overlapping CRMs was estimated by the number of changes in the TF binding profile (Hamming distance) and by the proportion of different TF binding sites (Jaccard distance). At the promoter regions, we observed less dissimilarity in CRMs between K562 and GM12878 than by chance (Figure 4). 62% of the overlapping CRMs have a difference in binding of one or two TFs between the cell types, and 63% of those contain only three or four TFs in K562. Interestingly, at the promoters of genes, which are differentially expressed between K562 and GM12878, there is a higher than expected proportion of overlapping CRMs which have no difference in TF binding. When we predicted the level of expression for these genes using the co-localized TFs, we observed a mean squared prediction error (MPSE) of 2.05 for expression levels in GM12878, but a MPSE of 7.95 for expression levels in K562. Although GM12878 and K562 have the same TFs co-localized at the promoters for some differentially expressed genes, it seems that only in GM12878 are the co-localized TFs showing a regulatory effect on the target gene.

**Figure 5:**
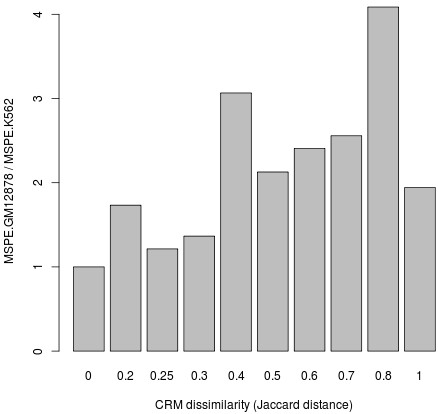
**Differences in expression prediction accuracy between dissimilar CRMs.** Different models, one generated from GM12878 CRMs and the other from K562 CRMs, were used to predict gene expression levels in the same cell type (K562). The ratio between the mean squared prediction errors of the GM12878 model (MSPE.GM12878) and K562 model (MSPE.K562) is higher for CRMs where a larger proportion of TF binding sites (Jaccard distance) differ between the two cell types.

The extensive dissimilarity in TF binding profiles at gene promoters between the two hematopoietic cell types could also influence differential expression. To examine the cell type-specific regulatory effect CRMs have on target genes, we compared the dissimilarity in TF binding between K562 and GM12878 to any difference in expression prediction accuracy. For each target gene, we constructed two expression prediction models which differ in their predictor terms. One model contains predictors which are co-localized TFs that bind to CRMs in the K562 cell. In the other model, the co-localized TFs are those that bind to CRMs detected in the GM12878 cell. Both sets of models were trained on the DMap expression data set of 38 distinct hematopoietic cell populations. We then assessed how well the two sets of models predicted target gene expression in K562. The models generated from GM12878 CRMs that are identical to K562 CRMs had a MSPE of 0.469. In contrast, a MSPE of 1.58 was observed for models generated from GM12878 CRMs that are 80% dissimilar in TF binding compared to their corresponding K562 CRMs. The models generated from GM12878 CRMs, where more than 50% of the TF binding sites are different from the corresponding K562 CRMs, have significantly higher expression prediction errors (p-value =1.6×10^−4^). There is no significant difference in expression prediction errors (p-value =0.681) if less than 30% of the TF binding sites are different. The general trend of expression prediction errors seem to be increasing for GM12878 CRMs that are more dissimilar to K562 CRMs (Figure 5). This suggests that prediction of gene expression is sensitive to the cell type-specificity of the TF binding profiles.

### TF co-localization patterns specific to differentially expressed genes

**Figure 6:**
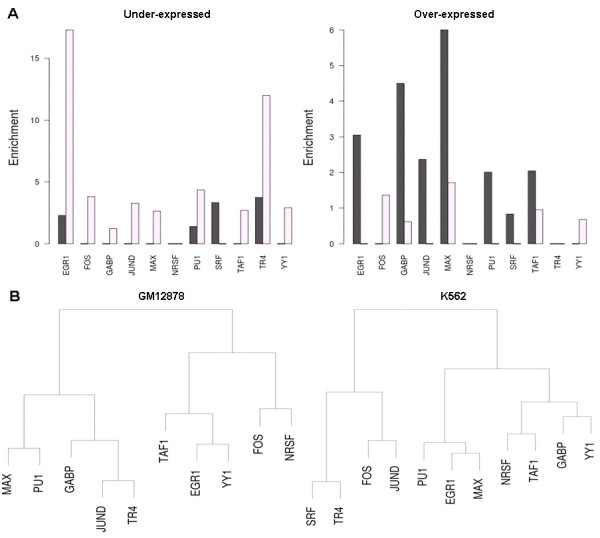
**TF binding differences between CRMs at differentially expressed genes.** (**A**) Dissimilarity of TF binding sites at the promoters of genes which are under-expressed and over-expressed in GM12878 compared to K562. We compared the enrichment (observed/expected numbers) of TFs that bind to CRMs in GM12878 but not in K562 (black bars) and TFs that bind to CRMs in K562 but not in GM12878 (white bars). (**B**) Hierarchical clustering of TFs based on co-occurrence at promoters of the differentially expressed genes. TFs are clustered closer together if their binding sites co-localize more often in the CRMs of K562 and GM12878.

**Figure 7:**
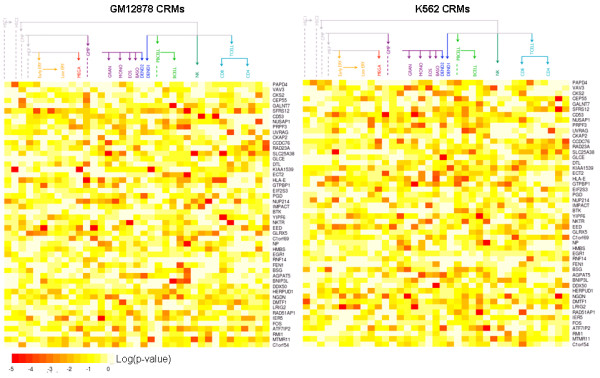
**Expression prediction accuracy for top 50 most variable genes across hematopoietic cell groups.** Significance of expression prediction accuracy across hematopoietic cell types for K562 and GM12878 CRMs. We used CRMs to predict the expression of each gene in a cell type and calculated the mean squared prediction error from the observed expression. To test whether the prediction error is significantly low, we found the probability (p-value) of obtaining a prediction error just as low from using randomly generated CRMs to predict expression. Only the differentiated cell types are column labeled; left of each label are the columns for their progenitors.

The differential expression between genes in K562 and GM12878 seems to correspond to the occurrence of specific TFs in CRMs (Figure 6A). Genes that are under-expressed in GM12878 compared to K562 have a higher than expected number of TFs binding promoters only in K562. In contrast, genes expressed higher in GM12878 compared to K562 have a higher than expected number of TFs binding to promoters only in GM12878. The absence and presence of TFs that correlate with over-expression and under-expression of genes may indicate the role of these TFs as activators or repressors. This is most apparent for *EGR1*, *GABP*, and *MAX*. However, TFs interact with other factors in CRMs, so the main effect of a TF is difficult to define. We found that different factors co-localize with *YY1* and *FOS* in K562 and GM12878 cells (Figure 6B), and this could explain why binding by those TFs are associated with both over-expression and under-expression of genes. When we compared how well CRMs detected in K562 and GM12878 predicted the expression of the top 50 genes with the most variable expression across hematopoietic cell types, neither K562 nor GM12878 CRMs had significantly high prediction accuracy across all cell types (Figure 7). Despite this, we still see that some K562 and GM12878 CRMs can accurately predict the expression of genes in some cell types. In the case of a possible tumor suppressor, *BNIP3L*, the combination of TFs *FOS* and *PU1* binding to the gene’s promoter (as observed in GM12878 cells) predicted the gene’s expression accurately only in B-cells. Differences in the prediction accuracy of a gene exist even between very similar cell types. For example, the expression of another possible tumor suppressor, *LRIG2*, in early-stage erythrocytes and monocytes is accurately predicted (p-value <0.01) using K562 CRMs, but its expression in late-stage erythrocytes and monocytes is not predicted accurately. When we compare prediction accuracy of K562 CRMs to GM12878, we see that *LRIG2* is better predicted by the K562 CRM across most cell types (Additional file 1: Figure S6A). This could be explained by the missing *EGR1* binding site and the additional *FOS*, *TAF1*, and *YY1* binding sites in the K562 CRM compared to the GM12878 CRM (Additional file 1: Figure S6B). Given that K562 cells belong more to the myeloid lineage and GM12878 cells belong more to the lymphoid lineage of blood cell development, it is surprising that there is no indication of K562 or GM12878 CRMs predicting expression accurately across genes in their respective blood cell lineages.

### Non-linear interactions between TFs and target genes

**Figure 8:**
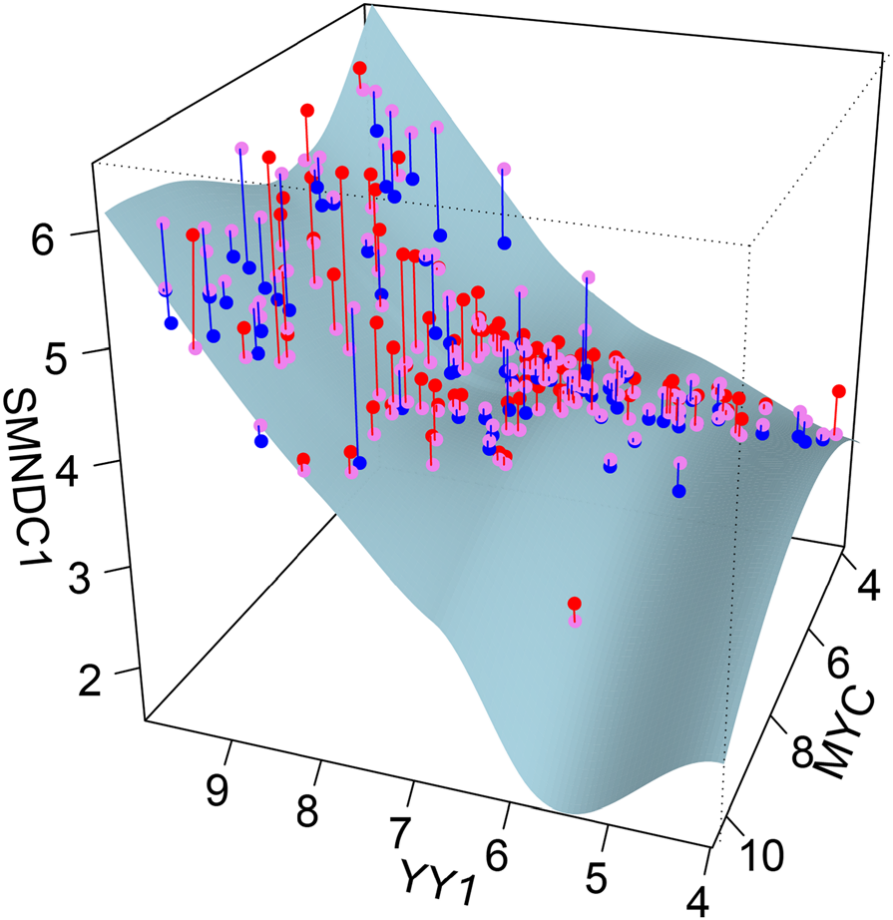
**3D plot of correlation between TF and target gene expression.** Regression plane of TF expression (MYC and YY1) against target gene SMNDC1. Repression activity of MYC and the activation activity of YY1 are captured in our model. The observed expression values (pink) are plotted on the surface along with their corresponding predicted expression values (blue if below, or red if above the observed values).

It is necessary to understand the combinatorial relationships between TFs given the high co-localization of TFs in the promoter regions. An advantage of modeling each target gene’s expression using a specific CRM is that we can describe the relationship between the target gene and multiple TFs. Of the 6582 total K562 CRMs tested, 2938 CRMs (45%) modeled using non-linear spline functions on the TF predictors fit significantly better (p-value <0.01) compared to linear models. The TF-target expression profiles (Additional file 1: Figure S7) clearly illustrates the non-linear relationship that target gene, *SMNDC1*, has with its TF predictors, *MYCMAX*, and *YY1*. Our model uses *MYC* as a predictor, which is reassuring considering that *MYC* is known to form a complex with *MAX*[28]. Furthermore, the U-shaped expression profile of *MYC* when plotted against *SMNDC1* suggests that *MYC* has divergent roles in different tissues. This divergent role of TFs not only appears in different tissues, but also in different genes. The smoothing functions from our models suggest that TFs like *MYC* and *MAX* may have both repressor and activator functionalities depending on their target gene (Additional file 1: Figure S9) . It is interesting to note that *YY1* and *MYC* also seem to interact differently with the same target gene (Figure 8). Non-linear regression allowed us to incorporate TF-TF interactions into our expression prediction models, however, of all the CRMs we examined in this study, only 2.2% of them have significant TF-TF interaction terms when used in models to predict target gene expression.

## Discussion

In summary, we have identified and characterized various regions of co-localized TFs in human K562 and GM12878 cells. The difference in co-localization patterns between GM12878 and K562 cells explains the differential expression in genes that may determine cell states. Dynamic CRM occupancy have been suggested as a regulator of temporal gene expression during cell development, but it cannot be inferred from TF binding motifs or static expression values [29]. Subsequently, our gene expression models were unable to predict with high accuracy the gene expression profiles of cell types that do not arise from a lineage similar to that of K562. The systematic analysis of CRMs dissimilar between K562 and GM12878, demonstrated that K562 gene expression could not be explained using GM12878 CRMs if its TF binding profiles are too dissimilar from that of the K562 TF binding profiles. Nevertheless, we found several cases where TF combinations detected in K562 or GM12878 CRMs could be used to predict gene expression in other cell types. Genes for which we know little about, like *LRIG2*, but associated with poor prognosis of different cancer types [30], have been detected by our method to correlate with the expression of three TFs (*FOSTAF1* and *YY1*) across blood cell groups.

This study assumes that expression levels of the TFs explain less target gene expression if their binding locations are found further away from the target gene’s TSS. This could be justified by the decreasing affinity and regulatory influence on the target gene as the CRM is positioned further away from the TSS [11,31]. The observed decrease in prediction accuracy by CRMs further than 1kb from the TSS is the reason for why we considered only CRMs proximal to the target gene when predicting gene expression in a single cell sample (Additional file 1: Figure S8). Nevertheless, more distal CRMs can also have a large effect on target gene expression if DNA is folded in a way that distant elements can interact [32,33]. A more complete expression prediction model may be to include co-localized TFs which also bind distal to a target gene.

The TFs examined in this study are involved in many processes and are co-expressed with many different genes. This means that the high prediction accuracy may be due to the by chance selection of TFs which are co-expressed with the target gene. This could result in over-fitting of the models, therefore, it was important to compare the prediction accuracy with null models. We selected CRMs which describe target gene expression with high accuracy across cell types, and examined features of those models that are indicative of TF binding position. We found that most of these CRMs contain a high number of co-localized TFs, some of which are known to form heterodimers. For instance, pairs of TFs known to form heterodimers, such as *MYC-MAXNFYA-NFYB*, and *FOS-JUN*, co-occur in many of the detected CRMs. Target genes whose expression is not accurately predicted and have a low number of co-localized TF predictors in their promoters may be better modeled by nucleosome occupancy signals, or other epigenetic determinants [34,35]. These other signals may also explain some of the spatial variability in our expression predictions. For instance, detection of open chromatin in the promoter region of a gene may indicate more TFs that bind to the region and regulate the gene [36].

In this study, we demonstrate that many of the highly predictive expression models describe non-linear interactions between TFs and target genes. This suggests that many TFs have divergent roles in regulating gene expression, for example, a TF may contribute to activating a gene in one cell type but repress it in other cell types. When we modeled the gene expression of *SMNDC1*, its TF regulator *MYC* is shown to have repression activity only during low levels of *YY1*. Although we detected co-occurring binding sites for both *MYC* and *YY1*, our expression model suggests that *YY1* silences the repression activity of *MYC*. This is consistent with recent findings showing that *YY1* negatively regulates *MYC* activity in certain tumour cells [37]. Another example of negative regulation between TFs is the repression of *FOS* by *YY1*. Of the 1422 CRMs detected which contain both *FOS* and *YY1* binding sites, only one of these CRMs significantly predicted target gene expression. The under-representation of possible regulatory modules containing both *FOS* and *YY1* may be due to the transcriptional repression of the *FOS* gene by *YY1*[38]. This repression activity of other TFs may explain why we cannot clearly define the effect of *YY1* on differentially expressed target genes. Although not captured in our models, the repressor activity of *YY1* is regulated through acetylation by *p300* and *PCAF*[39]. The lack of post-translational information in our models represents a limitation in the inference of TF activity.

## Conclusion

Our simple method of modeling gene expression based on the expression of co-localized TFs have shown that cell type-specific TF binding information is required for determining cell type-specific gene expression. However, by modeling each gene individually, we have shown that TF combinations detected in one cell type could in some cases predict gene expression in other cell types. This could suggest possible regulators of a gene in a cell type for which we do not have TF binding data. Further extension of this basic approach may be needed to account for TFs that bind to non-genic regions and interact over a large genomic distance due to tertiary chromosome structure [40]. This requires integration of additional genomic markers for identifying target genes of distal regulators. Since we expect more cell type-specific data to be generated on TF binding and chromatin structures, our perspective of dynamic CRM occupancy will only become more complex. Therefore, we hope that this approach of integrating co-localized TFs to predict target gene expression will provide a useful way of capturing the combinatorial effect of TFs in different human cell types.

## Abbreviations

CRM, Cis-regulatory module; TF, Transcription factor; TSS, Transcription start site; MSE, Mean squared prediction error.

## Competing interests

The authors declare that they have no competing interests.

## Authors’ contributions

DW, WO, and LW conceived the study. DW, AR, and LW designed the experiments. DW conducted the experiments and performed the analysis. DW wrote the manuscript. All authors read and approved the final manuscript.

## Supplementary Material

Additional file 1: **Supplementary methods, figures and tables.** Contains additional information on methods, supplementary figures 1–9 and supplementary tables 1–2. Click here for file

Additional file 2: **Supplementary data.** Contains additional information on supplementary data set of CRM locations. Click here for file
