# Supplementary material for: Transcription factor co-localization patterns affect human cell type-specific gene expression
Source: BMC Genomics. 2012 Jun 21;13:263. doi: 10.1186/1471-2164-13-263 (PMC3441573; doi:10.1186/1471-2164-13-263)
Supplement: Additional file 1: — Supplementary methods, figures and tables. Contains additional information on methods, supplementary figures 1–9 and supplementary tables 1–2. [file 1471-2164-13-263-S1.pdf]

Additional Methods

Cell Expression Profiles

The tissue-dependent gene expression dataset from the Genome Novartis Foundation contains 32 healthy major tissues, and 47 tumour samples and cell lines. The custom-designed whole-genome gene expression microarrays used on each sample targets 44775 human mRNA transcripts. Previous analysis of this dataset identified many chromosomal regions of correlated transcription that are under the control of both tissue and parental allele-specific expression. The expression levels of TF genes across tissue samples are observed to be lower than non-TF genes. This is coherent with the mechanistic explanation that the effect of a single TF molecule is amplified by transcribing many copies of mRNA from a target gene. Across all samples, the proportion of TFs relative to all expressed genes is remarkably stable at $\sim 6\%$. In the bootstrap test for highly predictive CRMs, we resampled from this set of TFs to generate the bootstrap replicates. High variance in gene expression profiles are observed between replicates for samples with more heterogeneous composition. Therefore, we treat each replicate as an independent sample in our analysis. When analyzing expression variation in a single sample, we found that a Gaussian distributional assumption for gene expression is more suitable compared to other distributions.

Smoothing and Model Fitting

Since gene expression response by the target gene varies over different TF expression values in a smooth fashion, a curved function is needed to fit our gene expression data. For additive models, the partial response of the target gene to the expression of each TF is described by a smooth function. The smooth functions specified by GAM are scatterplot smoothers such as loess (a locally weighted regression smoother), running mean, or a smooth spline. The specific scatterplot smoother used in this application of the GAM is the penalized spline. The penalized spline s can be written in terms of basis functions thus:

$$s(x) = \sum_{i=1}^{k} \beta_{i+1} b_i(x)$$

where k is the number of basis functions used for s, the β_i is the regression coefficients to be estimated, the b_i are the basis functions, and x is the log expression value of a TF in the CRM. If we let $x_j^* : j = 1...k$ be a set of points (knots) in the range of x, the smoothing function $s(x)$ amounts to using sections of cubic polynomial joined at the knots. The knots in effect control the degree of smoothing for the curve. This is achieved by representing the basis functions as $b_j(x) = |x - x_j^*|^3$. The number of basis functions should be large enough to be able to approximate the true relationship between TFs and target gene. However, since the gene expression data is inherently quite noisy, it is likely that the model will overfit data if the number of basis functions is too large. This problem is alleviated by using a relatively large number of basis functions but avoiding overfit by imposing a penalty during model fitting.

To describe the fitting procedure, consider fitting a gene expression prediction model with two TF predictors to Gaussian target gene expression data with an identity link.

The fitting objective for our gene expression prediction models will be to minimize:

$$\sum_{i=1}^{n}(y_i - \beta_0 - s_1(x_{1i}) - s_2(x_{2i}))^2 + \lambda_1 \int [s_1''(x_1)]^2 dx + \lambda_2 \int [s_2''(x_2)]^2 dx$$

where i is each condition and λ is a smoothing parameter that controls the trade-off between good fit and model smoothness. The penalty is applied to the wiggliness of each smoothing function, as measured by $\int [s''(x)]^2 dx$. The fitting procedure for GAM follows the same method of iteratively reweighted least squares (IRLS) for generalized linear models, but we estimate the mean squared error (MSE) of the fitted model as a function λ using cross-validation. In each cross-validation step, we leave out an expression value, fit the model to the remaining expression values, and calculate the squared difference between the left out datum and the fitted model. The calculation is repeated for each expression value of the target gene or sample, and hence we obtain the average squared difference between missing data and the model fitted to the remaining data. A computational shortcut for the n-fold cross-validation is to use the Generalized Cross Validation (GCV) score. The GCV is achieved by writing the cross-validated MSE as a weighted sum of the model residuals and by replacing all the individual weights in the summation by the average weight. Subsequently, the trace of the influence matrix in the score is the estimated degrees of freedom for the model.

Additional Tables and Figures

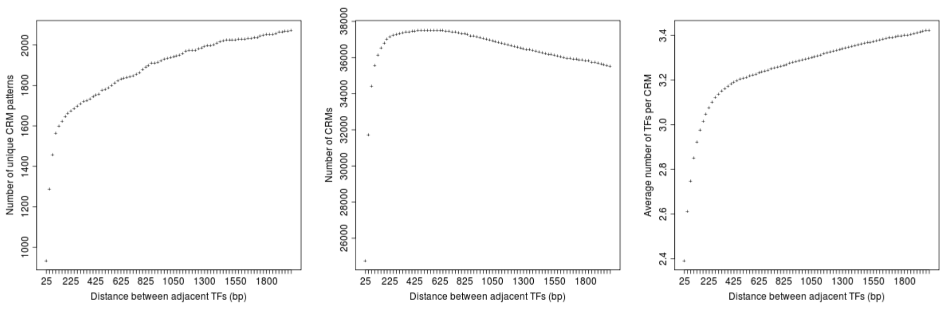

Figure 1: Distance between co-localized TFs. Size and number of CRMs obtained for different distance requirements between adjacent TFs. We defined adjacent TF binding sites to be co-localized if they are within 500bp of each other.

Table 1: List of TFs assayed by ChIP-Seq and downloaded from ENCODE

Cell	TF	Description
K562, GM12878	EGR1	Early growth response protein 1 (Nerve growth factor-induced protein A)(Zinc finger protein 225)
K562, GM12879	FOS[a]	Proto-oncogene protein c-fos (Cellular oncogene fos)(G0/G1 switch regulatory protein 7)
K562, GM12880	GABP	GA-binding protein alpha chain (GABP subunit alpha)(Nuclear respiratory factor 2 subunit alpha)
K562, GM12881	JUND[a]	Transcription factor jun-D (activator protein 1)
K562, GM12882	MAX[a]	Protein max (Myc-associated factor X)
K562, GM12883	NRSF	RE1-silencing transcription factor (Neural-restrictive silencer factor)(X2 box repressor)
K562, GM12884	PU1	Transcription factor PU.1 (31 kDa-transforming protein)
K562, GM12885	SRF	Serum response factor (c-fos serum response element-binding transcription factor)
K562, GM12886	TAF1	Transcription initiation factor TFIID subunit 1 (Cell cycle gene 1 protein)
K562, GM12887	TR4[a]	Nuclear receptor subfamily 2 group C member 2 (Testicular receptor 4)
K562, GM12888	YY1[a]	Transcriptional repressor protein YY1 (Yin and yang 1)(INO80 complex subunit S)
K562	NFYA[a]	Nuclear transcription factor Y subunit alpha (CAAT-box DNA-binding protein subunit A)
K563	NFYB[a]	Nuclear transcription factor Y subunit beta (CAAT-box DNA-binding protein subunit B)
K564	JUN[a]	Transcription factor AP-1 (Activator protein 1)(Proto-oncogene c-jun)
K565	MYC[a]	Myc proto-oncogene protein (c-Myc)(Transcription factor p64)
K566	GATA1[a]	Erythroid transcription factor (Eryf1)(GATA-binding factor 1)(NF-E1 DNA-binding protein)
K567	ZNF263[a]	Zinc finger protein 263 (Zinc finger protein with KRAB and SCAN domains 12)
K568	GATA2[a]	Endothelial transcription factor GATA-2 (GATA-binding protein 2)
K569	NFE2[a]	Transcription factor NF-E2 45 kDa subunit (Nuclear factor, erythroid-derived 2 45 kDa subunit)
K570	SIRT6[a]	Mono-ADP-ribosyltransferase sirtuin-6 (EC 2.4.2.31)(SIR2-like protein 6)

[a]marks the 14 TFs used in the analysis of K562 CRMs.

Table 2: Proportion of gene expression variation explained by CRMs for each sample in the Novartis GNF microarray data-set. The table shows a listing of R^2 statistics between observed and predicted gene expression for each tissue and cell sample found in the Novartis GNF microarray data-set.

**Sample**	R^2
Occipital Lobe	0.386742784
Medulla Oblongata	0.350439766
BLymphoblasts	0.295023059
Brain Caudate Nucleus	0.283746596
Pons	0.254854625
PB CD8 T Cells	0.247515533
PB CD14 Monocytes	0.223050139
Cingulate Cortex	0.220783545
Leukemia Promyelocytic HL60	0.219273143
PB BDCA4 Dentritic Cells	0.201826195
Atrioventricular Node	0.199529434
PB CD56 NK Cells	0.19859689
Brain Amygdala	0.196190799

Continued on next page

Table 2 – continued from previous page

Sample	R^2
Subthalamic Nucleus	0.19421931
Leukemia Lymphoblastic MOLT4	0.181771555
Hypothalamus	0.175745236
Brain Thalamus	0.171753079
Globus Pallidus	0.162232745
PB CD19 B Cells	0.16192942
Prefrontal Cortex	0.158151741
BM CD33 Myeloid	0.157633054
BM CD34	0.156698154
DRG	0.155394141
Lymphomaburkitts Daudi	0.150770109
Temporal Lobe	0.10420283
Skeletal Muscle Psoas	0.102281928
Leukemia Chronic Myelogenous K562	0.101532493
Trigeminal Ganglion	0.096166697
Ciliary Ganglion	0.093846306
Cerebellum Peduncles	0.093475756
Parietal Lobe	0.087686383
BM CD105 Endothelial	0.087202579
Superior Cervical Ganglion	0.050121391
Appendix	0.049479716
Skin	0.042814378
Whole Brain	0.03884314
Lung	0.036190547
Fetal Brain	0.036103812
Adrenal Cortex	0.034661211
HBEC	0.031241613
Trachea	0.030727988
Uterus Corpus	0.02782047
Smooth Muscle	0.026992034
BM CD71 Early Erythroid	0.026687884
Cerebellum	0.024496838
Colorectal Adenocarcinoma	0.023368963
Placenta	0.021711552
Tongue	0.020882087
Fetal Lung	0.019803454
Lymph Node	0.018169224
Adrenal Gland	0.01769725
Uterus	0.016651643
Cardiac Myocytes	0.01534718
Bone Marrow	0.014970883
Olfactory Bulb	0.014125774
Lymphoma Raji	0.013767309
Thymus	0.0128992
Ovary	0.011391745

Continued on next page

Table 2 – continued from previous page

**Sample**	R^2
Thyroid	0.011216933
Prostate	0.010477776
Pituitary	0.010286725
Liver	0.009622452
Kidney	0.009296748
Testi Seminiferous Tubule	0.007643048
PB CD4 T Cells	0.007620555
Heart	0.007053728
Fetal Liver	0.004190705
Whole Blood	0.003715035
Pancreas	0.003397941
Testi Leydig Cell	0.002862611
Spinal Cord	0.002431528
Testi Germ Cell	0.001793374
Human Cultured Adipocyte	0.001515583
Testi Intersitial	0.001133993
Salivary Gland	0.000999109
Testis	0.000639887
Islet Cell	0.000481884
Fetal Thyroid	0.000143145
Tonsil	9.66594E-05

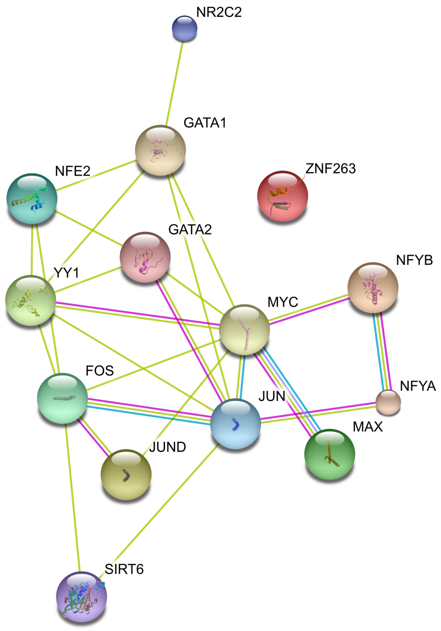

Figure 2: Protein interaction network between TFs from the String Database. The network is generated based on evidence from textmining (yellow), TF databases (blue), experiments (purple), and co-occurrence (dark blue).

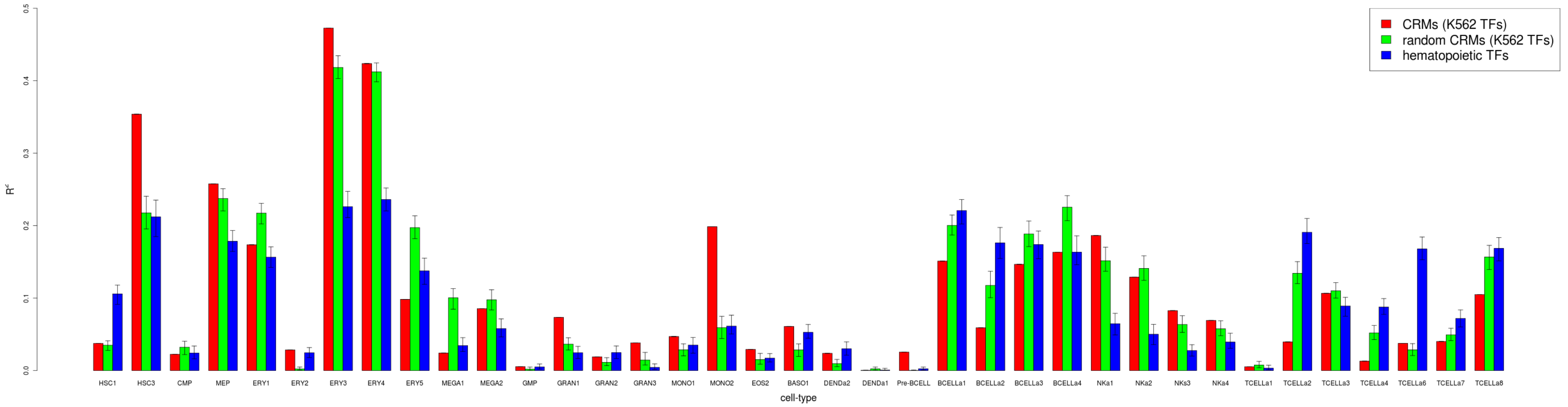

Figure 3: Comparing gene expression prediction accuracy across different hematopoietic cell types. Three different sets of predictors are used to predict expression: K562 TFs, randomly sampled K562 TFs, and 41 TFs enriched in expression modules regulating hematopoiesis. For terminally differentiated monocytes (MONO2), which are closely related to K562, the observed CRMs are much more predictive of gene expression than models where we do not consider TF co-localization patterns.

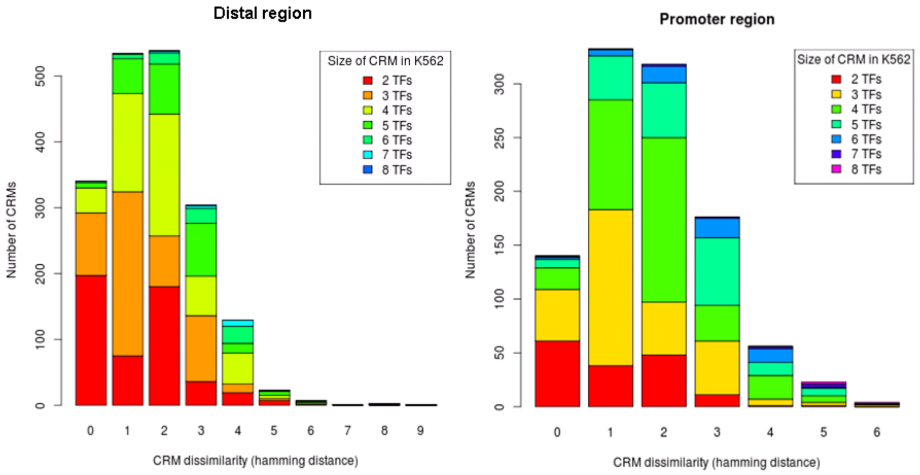

Figure 4: Stratified distribution of overlapping CRMs in the promoter (1 kb from TSS) and distal (>5kb from TSS) regions of the K562 and GM12878 genomes. Dissimilarity between an overlapping pair of CRMs is described by the number of different TFs (Hamming distance) bound to a K562 CRM compared to a GM12878 CRM. Stratification of CRM sizes (coloured bars) show that a high proportion of CRMs in K562 contain three or four different TFs, while the dissimilarity shows that most of the corresponding CRMs in GM12878 are different by one or two TFs.

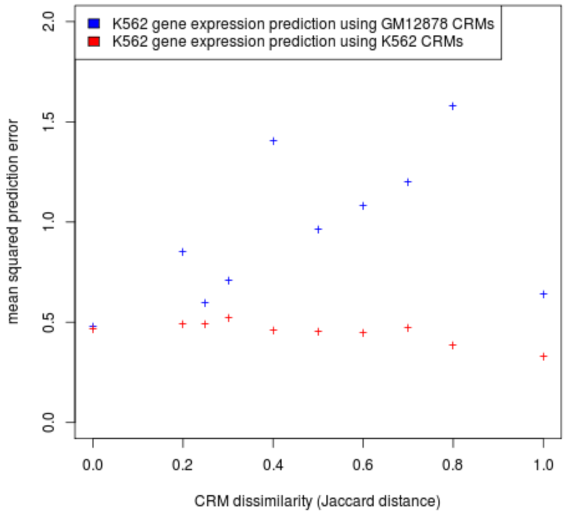

Figure 5: Expression prediction accuracy as a function of CRM dissimilarity. Prediction of gene expression levels in K562 cells was performed using models generated from GM12878 CRMs (blue) and K562 CRMs (red). The expression prediction error is higher for genes with CRMs that have a larger proportion of TFs (Jaccard distance) differing between the two cell types.

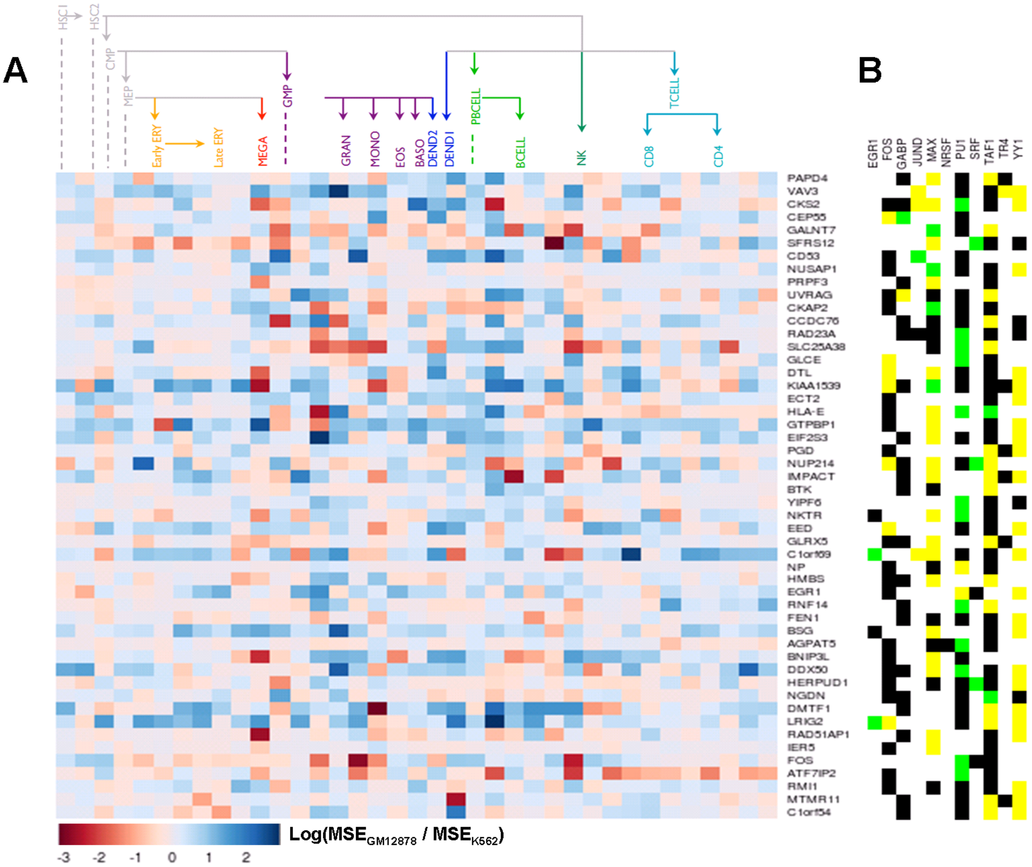

Figure 6: (A) Comparison of expression prediction accuracy across hematopoietic cell types using K562 and GM12878 CRMs. We predicted expression for 50 genes that have the highest variability in expression across cell types, but using different CRMs to inform the models. The expression of genes that are more accurately predicted by K562 CRMs for certain cell types are coloured more blue, while those more accurately predicted by GM12878 CRMs are coloured more red. Only the differentiated cell types are column labeled; left of each label are the columns for their progenitors. (B) TF occupancy of CRMs at the genes. TFs present in both K562 and GM12878 CRMs are marked in black, TFs present in only the K562 CRM are marked in yellow, and TFs present in only the GM12878 CRM are marked in green.

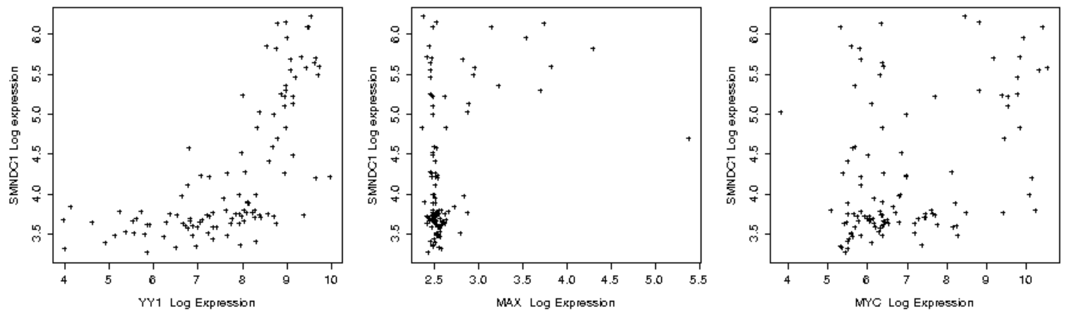

Figure 7: Expression profiles of three TFs in a CRM plotted against the expression profile of their target gene, SMNDC1. Each point is the log absolute expression value in a cell sample from the Novartis SymAtlas data set. Non-parametric smoothing functions in our model are used to describe these non-linear TF-target interactions.

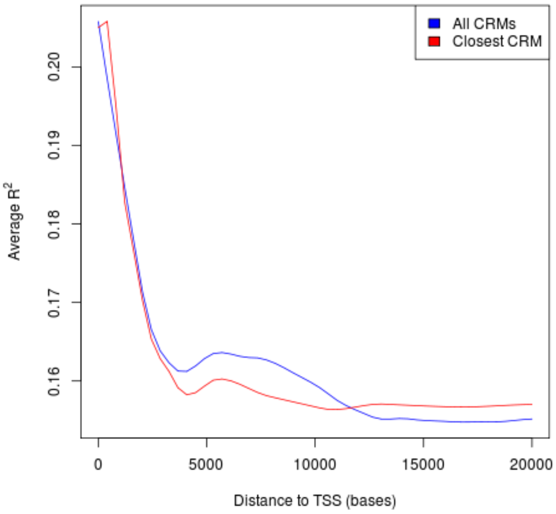

Figure 8: Assessment of the GAM model on gene expression prediction for different CRM positions relative to the TSS. The mean R^2 decreases as we consider CRMs further away from the TSS, but prediction accuracy does not decrease if the CRM described in the model is the closest to the TSS.

Figure 9: Component smooth functions that make up GAM models for the top 10 predicted genes (significantly higher R^2 compared to null models) are plotted below. Observed expression values for each TF is plotted against mean centered target gene expression and confidence bands are in red.

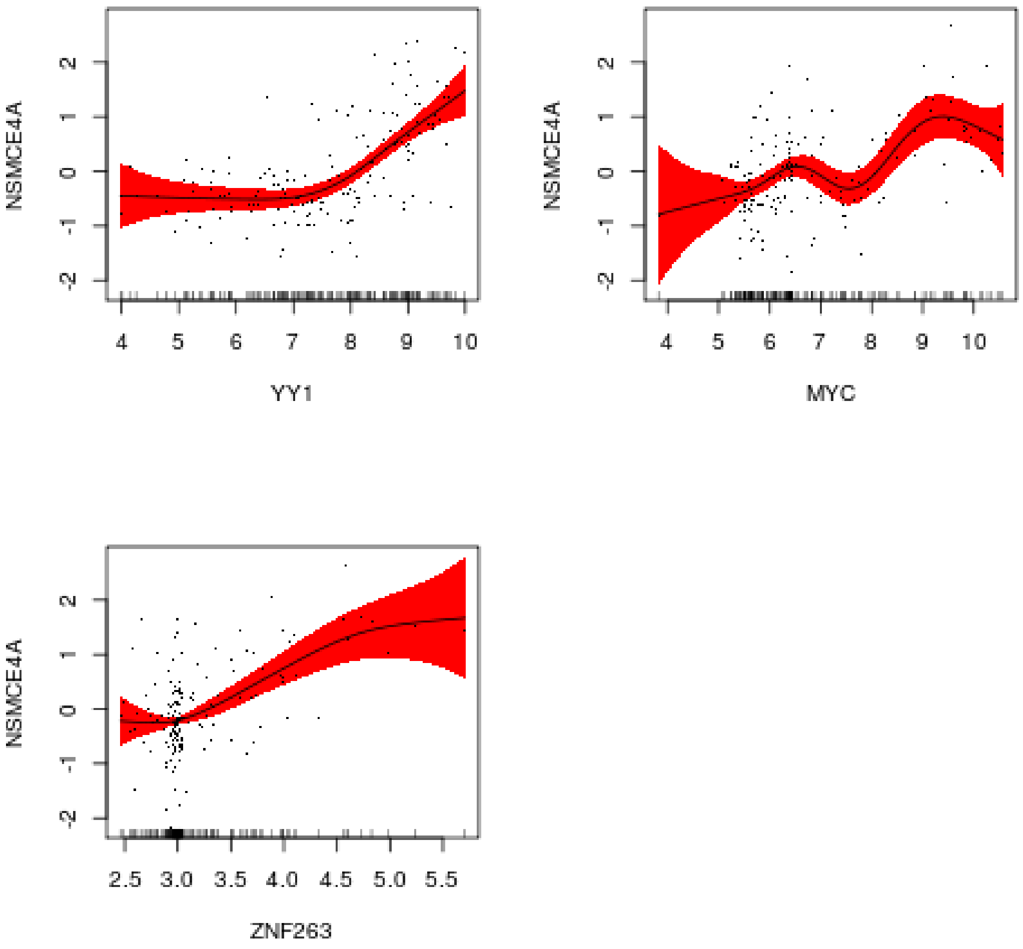

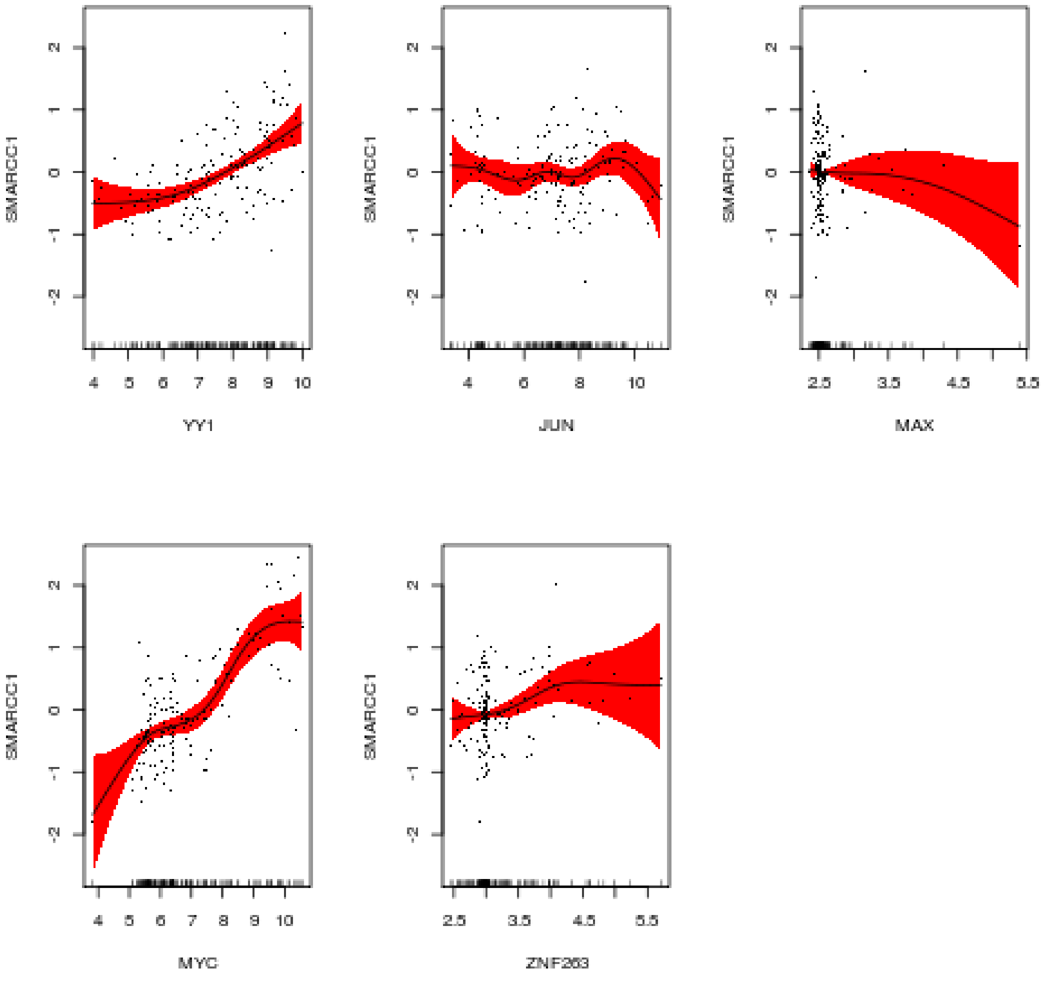
SMARCC1
YY1
JUN
MAX
MYC
ZNF263

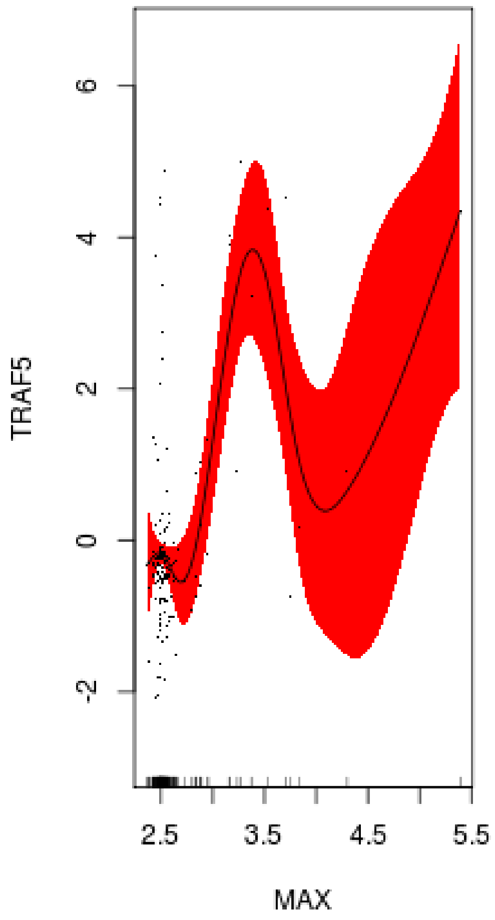

TRAF5
MAX
2.5
3.5
4.5
5.5
-2
0
2
4
6
TRAF5
MYC
4
5
6
7
8
9
10

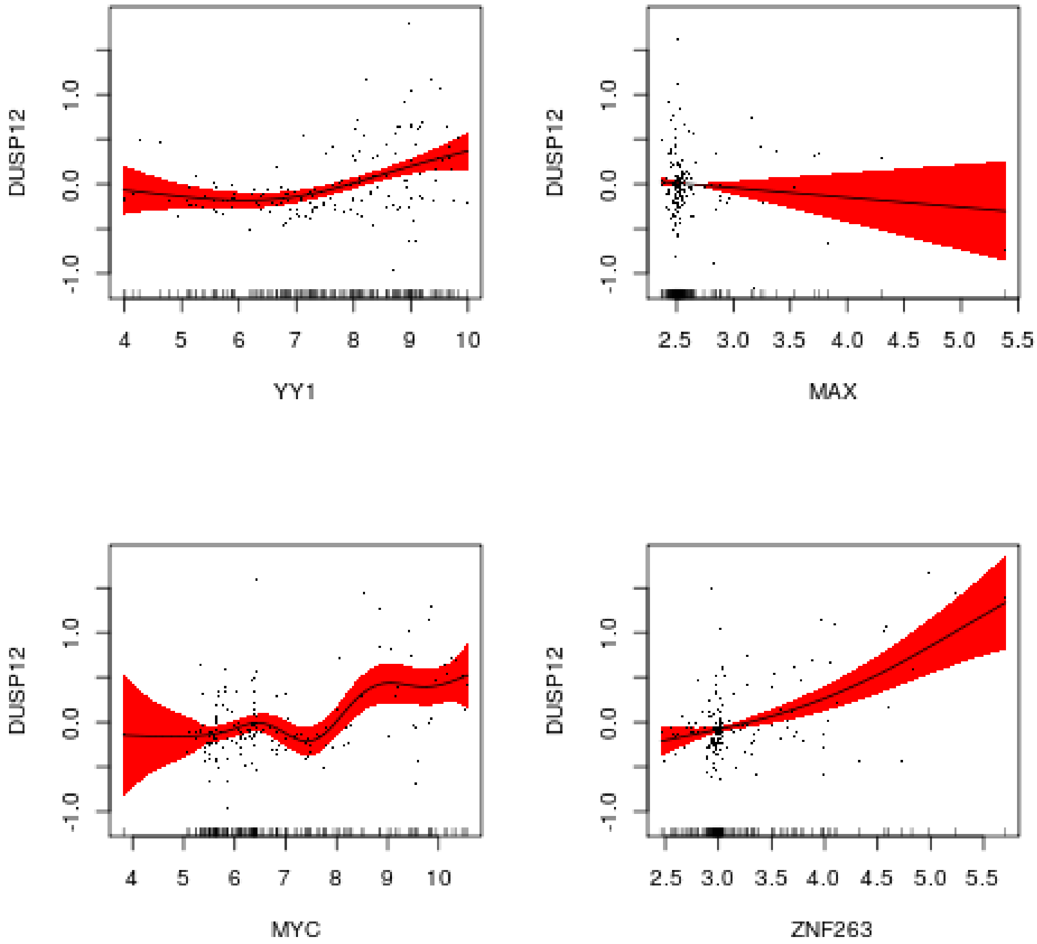
DUSP12
YY1
MAX
MYC
ZNF263
-1.0
0.0
1.0
4
5
6
7
8
9
10
2.5
3.0
3.5
4.0
4.5
5.0
5.5

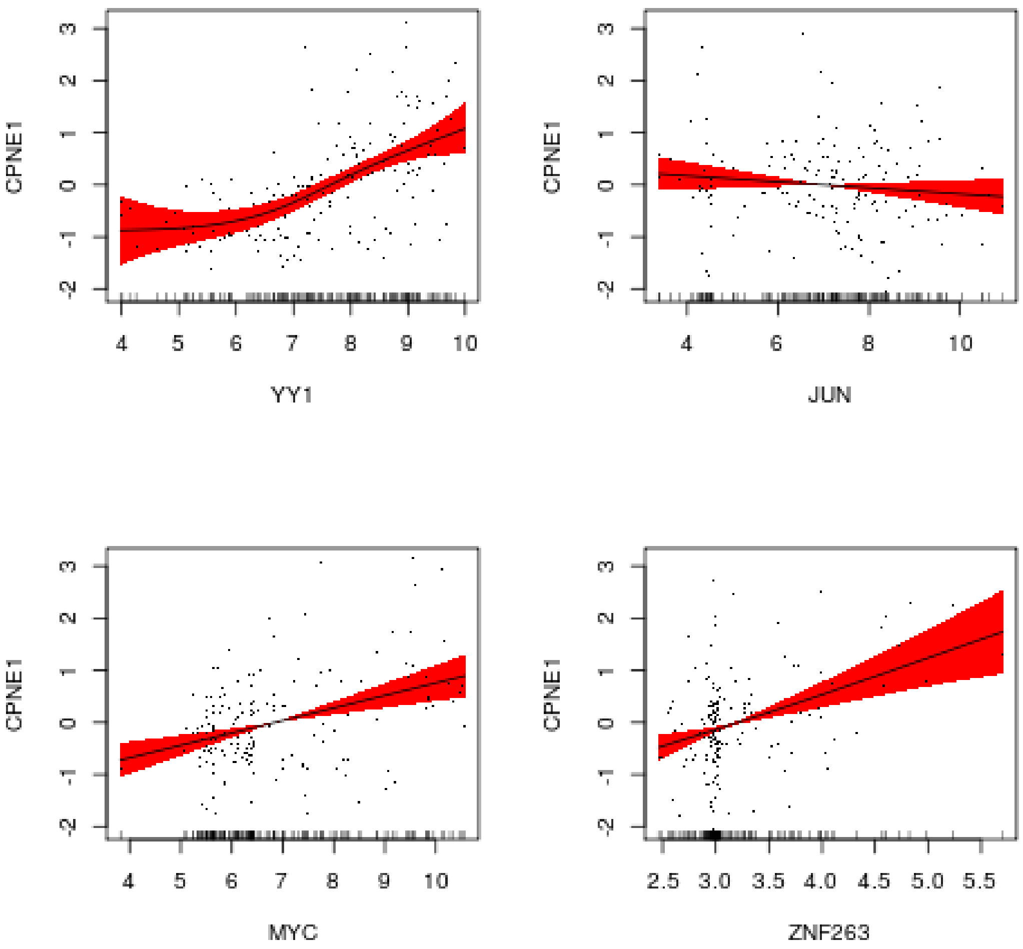

CPNE1
YY1
CPNE1
JUN
CPNE1
MYC
CPNE1
ZNF263

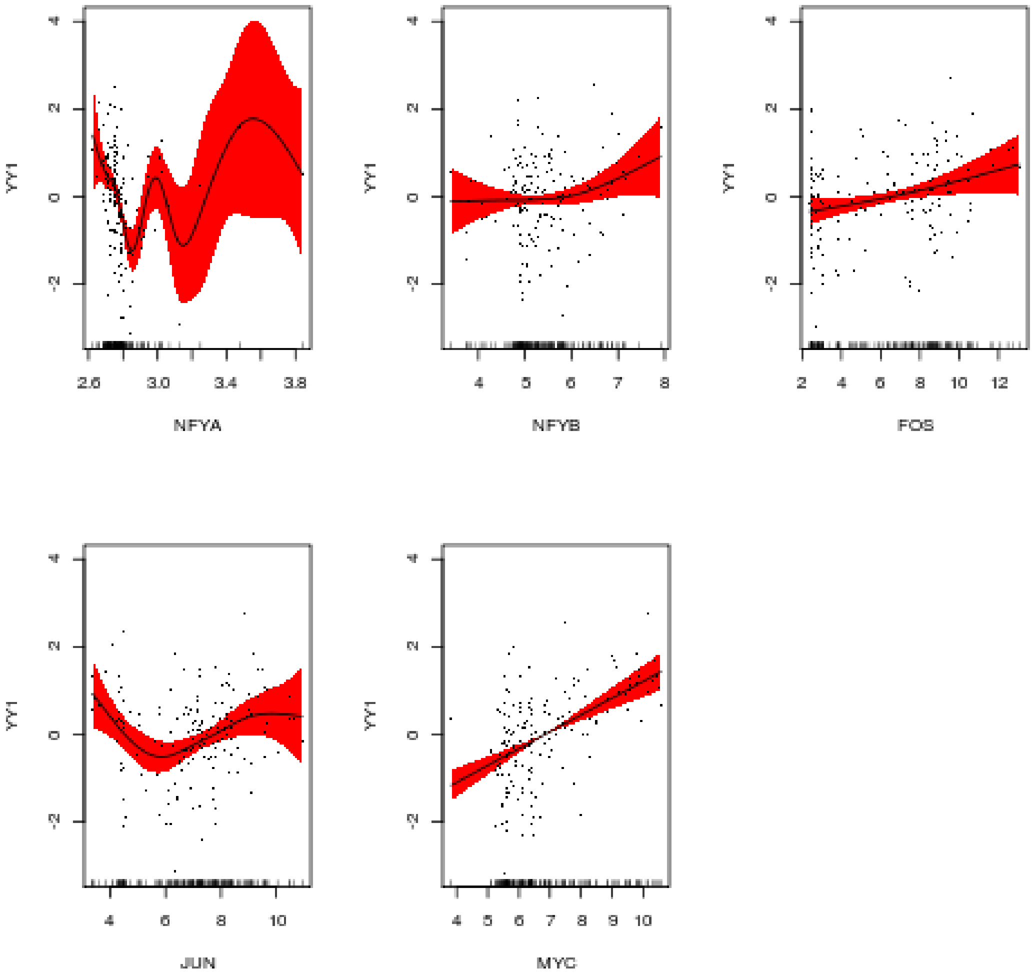
YY1
NFYA
NFYB
FOS
JUN
MYC

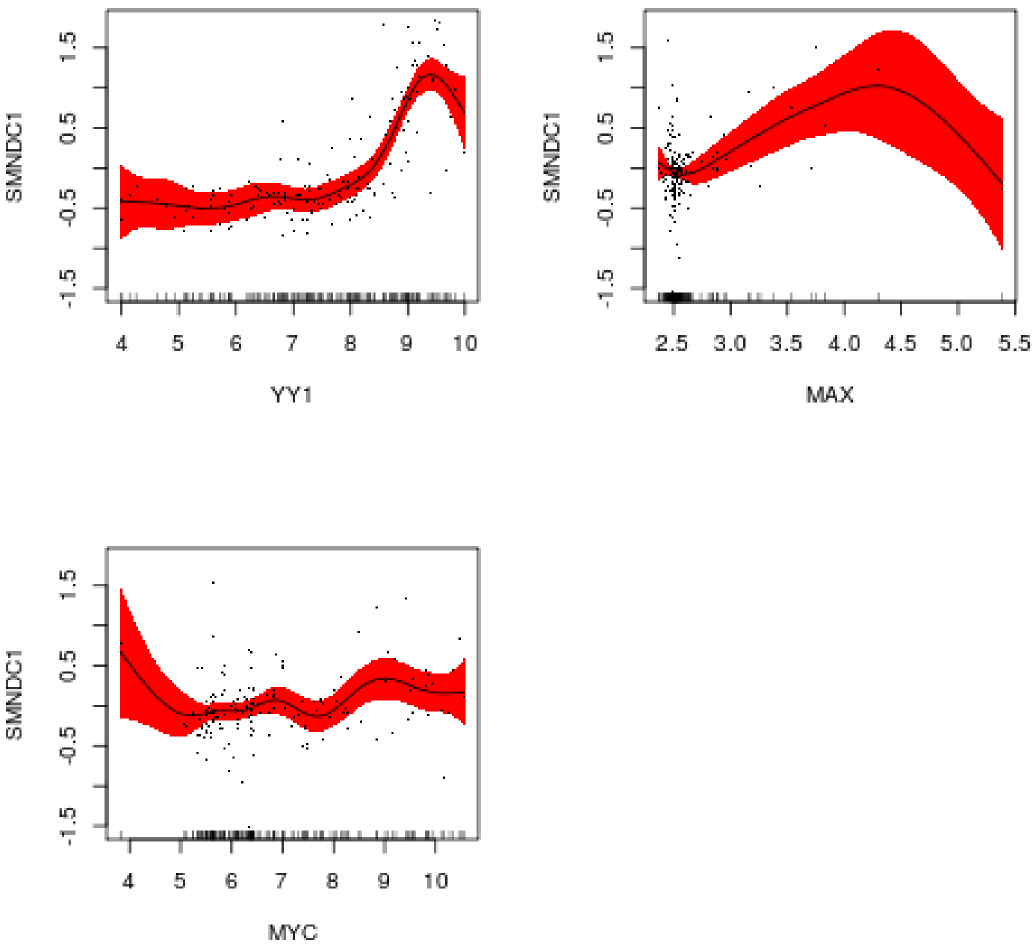

SMNDC1
1.5
0.5
-0.5
-1.5
4
5
6
7
8
9
10
YY1
2.5
3.0
3.5
4.0
4.5
5.0
5.5
MAX
MYC

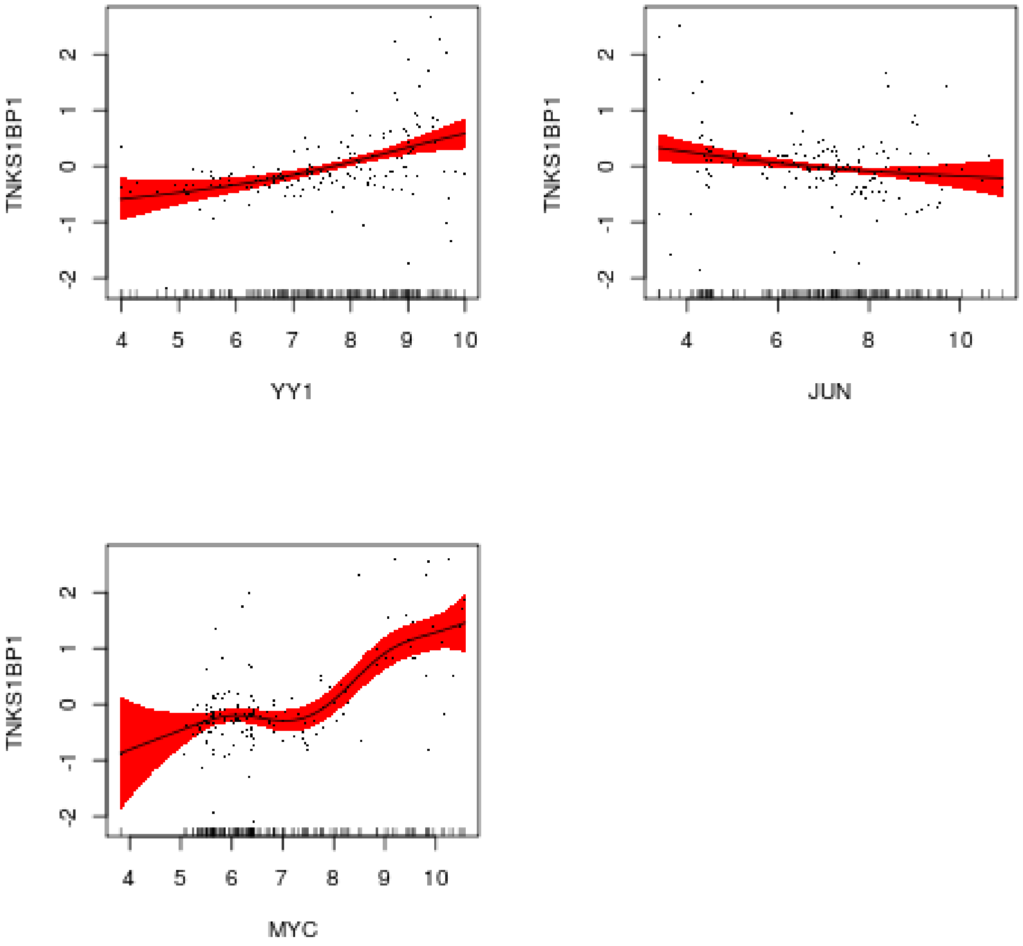
TNKS1BP1
YY1
JUN
MYC
-2 -1 0 1 2
4 5 6 7 8 9 10
4 6 8 10

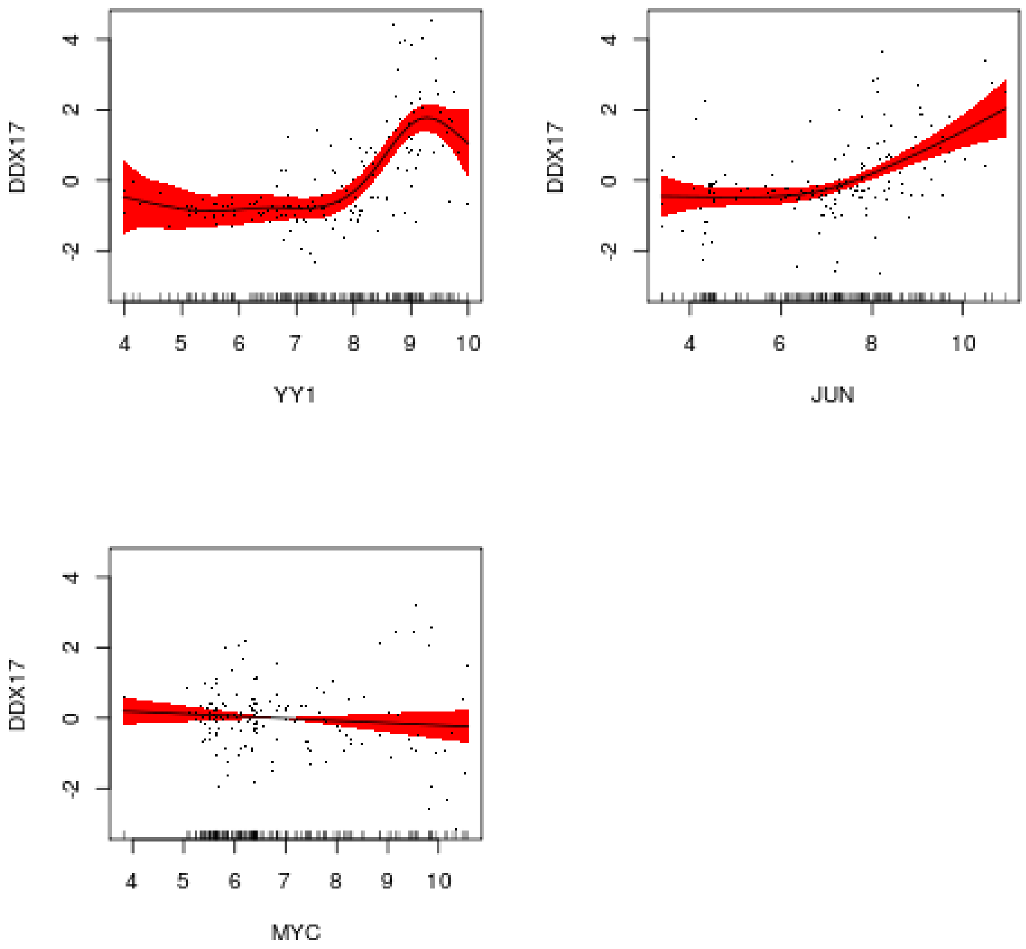
DDX17
4
2
0
-2
4
5
6
7
8
9
10
YY1
DDX17
4
2
0
-2
4
6
8
10
JUN
DDX17
4
2
0
-2
4
5
6
7
8
9
10
MYC
